# Applications of ultrasound to enhance fluidized bed drying of *Ascophyllum Nodosum*: Drying kinetics and product quality assessment

**DOI:** 10.1016/j.ultsonch.2020.105298

**Published:** 2020-07-30

**Authors:** Xianglu Zhu, Zhihang Zhang, Laura M. Hinds, Da-Wen Sun, Brijesh K. Tiwari

**Affiliations:** a Teagasc Food Research Centre, Ashtown, D15 DY05 Dublin, Ireland; b Food Refrigeration and Computerised Food Technology (FRCFT), School of Biosystems and Food Engineering, University College Dublin, Belfield, Dublin 4, Ireland

**Keywords:** Seaweeds, Airborne acoustics, Pre-treatment, Ultrasound, Fluidized bed drying, Drying kinetics

## Abstract

In this study, ultrasound either as a pretreatment technique or as an integrated technique was employed to enhance fluidized bed drying of *Ascophyllum nodosum*, and drying kinetics and dried product quality were assessed. In order to compare technology efficiency and dried product qualities, oven drying and fluidized bed drying (FBD) were employed. The novel drying methods included airborne ultrasound-assisted fluidized bed drying (AUA), ultrasound pre-treatment followed by FBD (USP), and hot water blanching pre-treatment followed byFBD (HWB). Six drying kinetics models were used to describe the drying curves, among which the Page model was the best in fitting USP and AUA. Model by Millidi et al. was employed to describe HWB. Airborne ultrasound in AUA did not reduce energy consumption or drying time, but retained total phenolic content (TPC) as well as colour, and exhibited the highest yield among the novel drying methods. USP and HWB showed lower energy consumption and drying time considerably, but the TPC was the lowest among the studied methods. At the same time, USP dried product exhibited the lowest *a_w_*, followed by HWB and then AUA. This studyalso demonstrated that FBD could be a very practical drying method on Irish brown seaweed, and ultrasound-assisted drying methods may have potential developments in Irish brown seaweed drying process.

## Highlights

• Novel fluidized bed drying methods saved seaweed drying time significantly.
• Airborne ultrasound assisted drying method retained highest total phenolic content.
• Page model was the best fitting ultrasound assisted fluidized bed drying kinetics.
• Seaweed drying after pretreatments saved more energy.

## 1 Introduction

Nowadays, the seaweed market scale all over the world is over 6 billion US Dollars per year, among which, 85% consists of food products and other 15% is contributed by seaweed extractions such as carrageenan, agar and alginates [1]. Seaweed is widely consumed and farmed as a low-calorie, nutritious food in many Asian countries especially in Korea, Indonesia, China and Japan [2]. Some of the edible seaweed referred to as ‘Kombu’ is sold in the dried form [3]. In the western countries, seaweed is normally regarded as a good resource of phycocolloids in the food processing industry such as agars, carrageenan and alginates [4]. In addition, seaweeds have been used as a fertilizer for centuries due to its high content of organic compounds such as amino acids, vitamins, proteins and manyother polysaccharides [5]. Many studies have proved that it can increase nutrient absorption and the growth rate of crops [6], [7]. Seaweed is a good source of biologically active phytochemicals and metabolites including fatty acids, polysaccharides, polyphenols, vitamins, minerals, meroterpenoids, etc. [8], [9]. These biologically active phytochemicals and metabolites may have potential treatment properties in a variety of diseases such as thyroid-related diseases, thrombosis, upper respiratory infection, tumour, obesity, diabetes etc. [8], [10], [11]. Seaweed can also beessential raw material for the biofuel, pharmaceutical and cosmetic industrie [12], [13], [14]. Due to the potential beneficial effects of various seaweed origin polyphenols on different cardiovascular-associated disorders and cancers such as hypertension, diabetes mellitus type 2, metabolic syndrome and breast cancer, total polyphenol content (TPC) is one of the key parameters to evaluate drying methods in present study [15], [16], [17]. North Atlantic rockweed (*Ascophyllum nodosum*) studied in the present paper, a typical brown seaweed is commonly harvested in northwest Europe, including Ireland, used for animal feed, fertiliser and alginate production [18], [19]. In order to optimise the use of all biological substances in fresh and perishable seaweedand extend shelf life of seaweed related products, an efficient preservation process is required prior to industrial or domestic seaweed use.

Drying is the most common method for the food industry [20], [21], [22], [23], which can also be used to further stabilize the biomass of fresh, harvested seaweed [24]. However, the quality of seaweed can be significantly affected by the drying process, especially when processed using high temperatures [25]. Low-cost methods of drying such as solar drying require a large space and a stable climate condition [26]. In Ireland, rotary dryers are commonly used for producing seaweed powder [27]. Some researchers have studied the effect on seaweed quality by different drying processes. Wong and Cheung [28] declared that oven drying is better than freeze-drying in terms of improving extractability and digestibility of protein in three seaweed species. Different drying methods were studied by Ling et al. [29],in which, oven drying and shade drying have shown better ability in retaining the biologically active phytochemicals in samples. Cruces et al. [30] claimed that freeze-drying is the best method of retaining the antioxidant activity of seaweed samples. Another factor to consider in this process is the energy consumption of drying technology. Among various drying methods, fluidized bed drying (FBD) offers many significant advantages such as high heat and mass transfer, high drying rate and even moisture reduction with less drying time. It can mix the entire solid product efficiently with drying air and provide with uniform drying temperature and longer constant drying rate period [31]. However hotspot formation in FBD dryers can result in a high moisture variation which may damage the product with loss of quality [31].

In order to develop a low-cost, efficient, drying technique with minimal impact to phytochemicals in seaweed, pre-treatments using ultrasound, microwave and osmosis have also been investigated [32], [33], [34]. Ultrasound, as a promising technique in the food industry has already shown the potential in accelerating freezeing, drying process, inactivating microbes, etc [35], [36], [37], [38], [39], [40], [41]. Pre-treatment operations such as hot water blanching, ultrasound, microwave etc, can modify the tissue structure and result in a shorter drying time [42]. In comparison with widely applied treatments such as hot water blanching, the non-thermal attribute of ultrasound can improve the final product quality and reduce the drying time [43]. The use of an ultrasound technique in the drying process canbeapplied in two ways: ultrasound pre-treatment before the drying process or airborne ultrasound irradiation during drying. Many studies have proven this technique to be efficient in relation to both drying time and product quality [44], [45], [46]. In terms of ultrasound pre-treatment in foods such as fruits and vegetables, drying time were shortened significantly in previous studies [47], [48], [49]. Ultrasound can also be employed directly during the drying process. Airborne ultrasound-assisted drying technologies also increases the water effective diffusivity while reducing the processing time. It was reported that drying time of zucchini, apple and strawberry was reduced by 13 to 44% [50], [51], [52]. Kroehnke et al. [53] even claimed an efficient hybrid convective drying method assisted by both ultrasound and microwave for carrot drying.

In order to investigate the effectiveness of fluidized bed drying combined with other novel technologies, hot water blanching, power ultrasound pre-treatment and airborne ultrasound combined with fluidized bed drying of *Ascophyllum nodosum* were explored in this study. Conventional oven drying, fluidized bed drying alone andcombinations offluidized bed drying techniques were conducted in order to compare various parameters of novel methods.

## 2 Materials and methods

### 2.1 Seaweed sampleand chemicals

Fresh *Ascophyllum nodosum* (moisture content of 73.08 ± 0.29%,w.b.) was harvested from the west coast of Ireland in November 2019. Fresh seaweed samples were washed thoroughly with tapwater to remove salt and surface impurities. Samples were then wiped with tissue to remove surface water and were then ground until approximately 1 to 2 cm in length. All samples were stored at −20 °C prior to further processing.

All reagents (ethanol, methanol, sodium carbonate, Folin-Ciocaltreau, gallic acid) were purchased from Sigma-Aldrich, AUA. Maximum recovery diluent CM0733 (MRD) and plate count agar CM0325 (PCA) were purchased from Oxoid, UK.

### 2.2 Drying methods

After defrosting the seaweedat 4 °C, 200 g of seaweed was used for each drying method.

#### 2.2.1 Oven drying

Conventional oven drying technique was carried out in using an oven (Gallenkamp Plus II, Gemini, Netherland), at 50 °C. The seaweeds were evenly placed in a tray (36 × 26 cm) in the oven.

#### 2.2.2 Fluidized bed drying (FBD)

Afluidized bed dryer (Sherwood Tornado M501, Sherwood Scientific, U.K.) was used to dry the seaweed samples. The drying temperature was set at 50 °C. The superficial velocity of hot air was 6.7 m/s. Initial loading height was about 9 ± 0.2 cm. The environmental relative humidity is 47.63 ± 2.51. Pulsed air flow was produced by the pulser inside fluidized bed dryer in order to homogenize the samples inside the dryer. The pulsing of the valve occurred in cycles of approximately 2.5 s closed and 2.5 s open. Samples were weighed every 10 min for the first 30 min of drying and every 15 min for the second 30 min, and every 30 min thereafter.

#### 2.2.3 Airborne ultrasound-assisted (AUA) fluidized bed drying

**Fig. 1:**
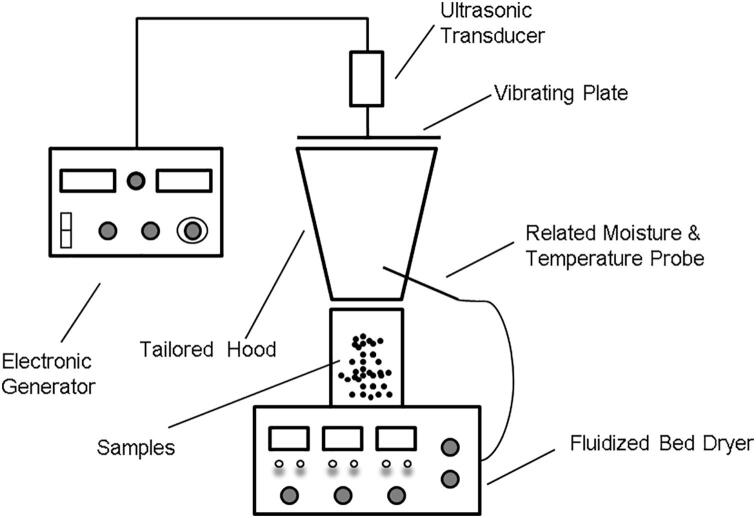
Experiment setup for airborne ultrasound-assisted (AUA) fluidized bed drying.

The AUA drying was conducted in an experimental setup combining the fluidized bed dryer aforementioned and an airborne ultrasound instrument (Pusonics S.L., Madrid, Spain), as illustrated in Fig. 1. A 26 kHz electronic wave was generated, amplified and transferred to the transducer, which connected to a horizontal vibration plate. Airborne ultrasound from the plate was transmitted downwards to the samples being dried by the fluidized bed dryer in the cylinder and conical hood. The temperature and velocity setting were the same as in FDB drying, while the power of the air-borne ultrasound was 170 W.

#### 2.2.4 Ultrasound pre-treatment (USP)followed by FBD drying

**Fig. 2:**
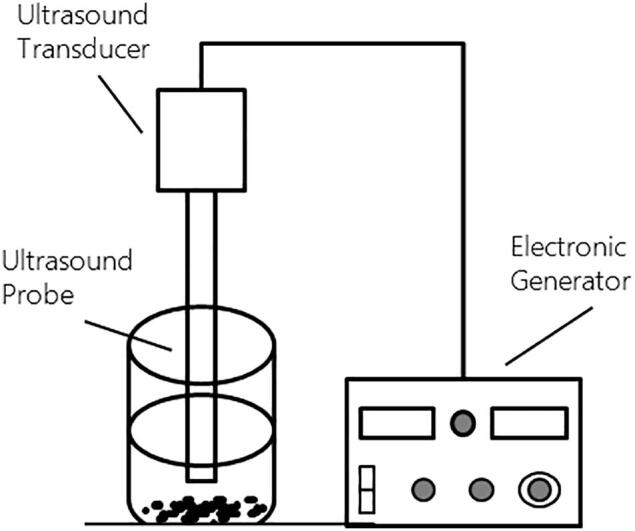
Experiment setup for ultrasound water bath pretreatment.

The samples were mixed with distilled water in a beakerat a ratio of 1:4 solid/water as recommended by Fernandes and Rodrigues [54] and Kadam et al. [55]. An ultrasound processer (500 W, UIP500hdT, Hislscher, Germany) at 20 kHz with a 13 mm diameter probe was employed in the ultrasound pre-treatment. The probe was submerged 30 mm under the water surface. The equipment configuration is shown in Fig. 2. After 10 min ultrasound pre-treatment at an amplitude of 100%, surface water was removed by vacuum filtering and blotting using a tissue. Thereafter, the samples were transferred to the fluidized bed for drying under the same drying conditions as for FBD drying.

#### 2.2.5 Hot water blanching (HWB) followed by FBD drying

The samples were blanched at 70 °C for 8 min in 800 ml distilled water in a beaker placed in a hot water bath (T100, Grant Instruments, U.K). Thereafter, the samples were filtered, then blotted to remove surface water, and finally dried as in FBD drying.

### 2.3 Moisture content measurement and electric power consumption measurement

Moisture content was evaluated by drying at 105 °C overnight in anoven (Model 28, Binder, Germany). Energy consumption of the various processes was determined, in terms of electric power consumption, using a power meter (PM 231E, Brennenstuhl, Germany). Total energy consumption measurement includes energy consumed at various stages of the drying process required to achieve 10% moisture content.

### 2.4 Colourimetric characterization

The colour was measured using a colourimeter (CR-400, Konica Minolta, Japan). It was calibrated with a white reference tile. Colour parameters of defrostednon-treated seaweed, as the control, were collected after washing and grounding into small pieces. After drying samples were wrapped with a film evenly and tested directly. Colour was expressed by means of CIE Lab coordinates (L*, a* and b*). Total colour difference (ΔE*) caused by the different drying methods was evaluated using Eq. (1). Defrosted sample before processing was taken as the reference.

(1) $$\Delta E^{*}=\sqrt{{\left(L^{*}-{L^{*}}_{r}\right)}^{2}+{\left(a^{*}-{a^{*}}_{r}\right)}^{2}+{\left(b^{*}-{b^{*}}_{r}\right)}^{2}}$$

where L* is lightness, a* is redness and b* is yellowness, subscript r indicates reference value from the defrost sample as a control. Eight replicates were conducted foreach sample.

### 2.5 Water activity (a*_w_*)

After calibration of standard solutions, a water activity meter (Series 3, AquaLab, USA) was used to measure water activity of samples. Measurements were performed at 23 ± 0.36 °C in three replicates.

### 2.6 Rehydration capacity

Ten grams of dried seaweed were immersed in 500 ml of distilled water kept at room temperature for 1 h. After rehydration, the seaweeds were drained using a wire mesh sieve and then blotted with tissue paper to remove surface water. The rehydration capacity of dried seaweed is estimated by the moisture content of the rehydrated sample.

### 2.7 Total phenolic content (TPC) measurement

Extractions of phenolic compounds were performed a method modified from Rajauria et al. [56]. Methanol (60%) was used as the extractant. Two grams of powdered seaweed samples, ground by a ball miler (MM 400, Retsch, Germany), were mixed with 20 ml methanol in dark conditions, shaking at 170 rpm at room temperature overnight. After filtration with a muslin cloth, the extract was concentrated using a nitrogen dryer at room temperature for 8 h, followed by freeze-drying. The freeze-dried samples were stored at −80 °C prior to further test.

Each freeze-dried extract was dissolved in water to prepare a solution at 0.8 mg/ml. The Folin-Coitreaunmethod, used by Ainsworth and Gillespie [57] and Ganesan and Bhaskar [58] with minor modification was employed in TPC evaluation in this study. Gallic acid standard solutions were used for calibration. After incubation with Folin-Coiltreau solution and sodium carbonate solution at dark for 30 min, the absorbance of the sample was recorded at 720 nm using a spectrophotometer (Epoch 2, Biotek, U.S.A.). Results are expressed as mg GA equivalents per gram dried seaweed sample.

### 2.8 Total viable count (TVC)

TVC evaluation was conducted based on ISO 4833-1:2013 [59]. Ten grams of samples were blended and diluted with MRD into different concentrations. One ml of each dilution was pipetted into a Petri dish. The pour plate method was applied using PCA as a growth medium. After 48 h incubation at 30 °C, colonies were counted and expressed as log CFU/gram. Three replicates were employed.

### 2.9 Scanning electron microscope (SEM) analysis

Dried seaweed samples were rehydrated as in Section 2.6 and immediately frozen in liquid nitrogen, followed by freeze-drying. The dried samples were mounted on stubs and then coated with a 5 nm layer of Gold by Emitech K575X Peltier Cooled Sputter Coating Unit (Quorum Technologies). Sample surfaces were photographed with a scanning electron microscope (Regulus 8230, Hitachi, Japan).

### 2.10 Mathematical modelling

In the drying process, the sample was weighed at fixed time points until a constant weight was achieved. Moisture content (w.b.) at any specifictime point (M_tw_) can be evaluatedby Eq. (2):

(2) $$M_{tw}=\frac{M_{0}∙W_{0}-{ML}_{t}}{W_{t}}$$

where M_0_, M_tw_ and ML_t_ are the initial moisture content (w.b.), moisture content and moisture loss at a given time. W_0_ and W_t_ are the initial sample weight (w.b.) and sample weight at a given time.

Moisture ratio (MR) was used for drying kinetics study, calculated as in Eq. (3)

(3) $$MR=\frac{M_{td}-M_{e}}{M_{0d}-M_{e}}$$

where M_0d_ and M_td_ are initial moisture content (d.b.) and moisture content at a given time, M_e_ is the equilibrium moisture content (d.b.). Six kinetic models shown in Table 1 were used forthe drying kinetics study.

**Table 1:** Six mathematical models applied describing drying kinetics.

Model	Equation	Reference
Newton	MR=exp(-kt)	Kumari and Khatkar (2018)
Henderson and Pabis	MR=a∗exp(-kt)	Touré (2019)
Page	$MR=\exp\left({-kt}^{n}\right)$	Yang et al. (2018)
Weibull	$MR=a*\exp\left({-kt}^{n}\right)$	Ju et al. (2018)
Midilli et al.	$MR=a*\exp\left({-kt}^{n}\right)+bt$	Midillietal. (2002)
Wang and Singh	$MR=1+at+{bt}^{2}$	Zhao et al. (2017)

### 2.11 Statistical analysis

Triplicates were performed for all drying and quality measurements unless otherwise stated. Pearson correlation analysis and principal component analysis (PCA) were performed to explore the correlation and correspondence between *aw* and dried sample colour using XLSTAT (version 2020.3, Redmond, Washington, USA). Effects of drying methods were evaluated using one-way analysis of variance (ANOVA) with Post HocTukey test, using SPSS (v20.0.0, IBM, U.S.A.). The significance level was defined as P < 0.05. Parameters of the six models were estimated using SPSS (v20, IBM, U.S.A.). The fitness of models was evaluated based on the sum square error (SSE; Eq. (4)), regression coefficient (R^2^), root mean square error (RMSE; Eq. (5)), chi-square (χ^2^; Eq. (6)), Akaike information criterion (AIC; Eq. (7)) and Bayesian information criterion (BIC, Eq. (8)).

(4) $$SSE=\frac{1}{N}\sum_{i=1}^{N}{\left({MR}_{expi}-{MR}_{predi}\right)}^{2}$$

(5) $$RMSE=\sqrt{SSE}$$

(6) $$\chi 2=\frac{\sum_{i=1}^{N}{\left({MR}_{expi}-{MR}_{predi}\right)}^{2}}{N-c}$$

(7) $$AIC=NIn\frac{SSE}{N}+2\left(c+1\right)+\frac{2(c+1)(c+2)}{N-c-2}$$

(8) $$BIC=NIn\frac{SSE}{N}+\left(c+1\right)In\left(c\right)$$

Where MR_exp_ and MR_pred_ are experimental and predicted moisture ratio, N represents the number of observations and c represents the number of constants in models.

## 3 Results and discussion

### 3.1 Drying kinetics, energy consumption and drying yield

**Fig. 3:**
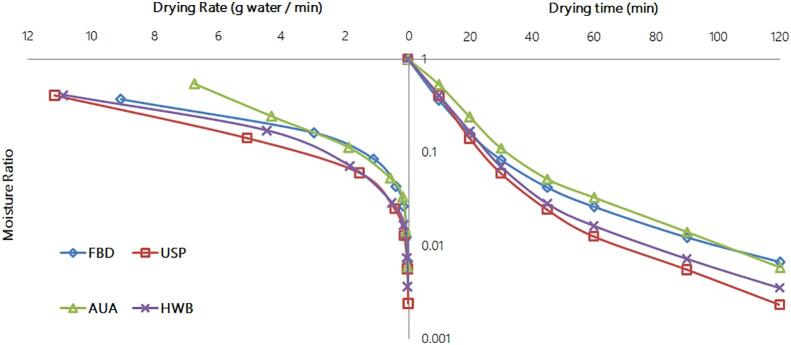
Drying curves and drying rate evolution for the fluidized bed drying (FBD), ultrasound pre-treatment assisted FBD (USP), airborne ultrasound-assisted FBD (AUA) and hot water blanching assisted FBD (HWB) Note: all data are the means from 3 replicates.

**Fig. 4:**
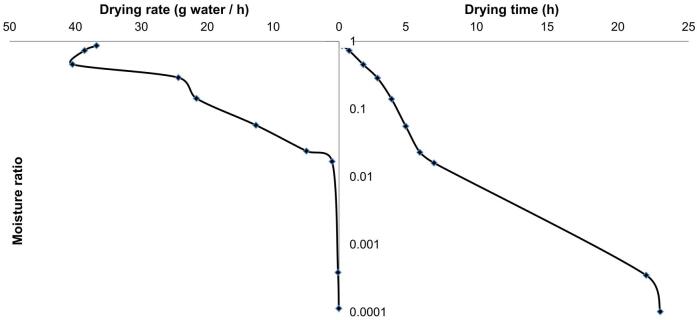
Drying curve and drying rate evolution for theoven drying *Note: all data are the means from 3 replicates.*

Drying rates and drying curves of the studied methods are shown in Fig. 3, Fig. 4. In a typical drying process, the drying rate goes through three stages: increasing, constant and decreasing stages [60]. The drying rate curve for the oven drying exhibited an increasing stage when the moisture content was still high at the beginning of the drying process and a subsequent fallingstagewhich was the predominant stage of the drying. However, in Fig. 3, all the other drying methods, which employed FBD drying, showed onlythe falling stage from the beginning. The falling stage as predominant drying stage for all the methods studied indicated that diffusion was the dominant mechanism for drying seaweed using FDB method. Similar results were also statedby Horuz et al. [61]. The occurrence of the increasing stage in oven drying was due to heating-up of the seaweed samples slowly in the oven. The other drying methods, where FBD drying was employed, could sharply heat up all seaweed sample particles in the bed. As a result, no increasing drying rate stage existed and their drying rates were much higher than the oven drying. Their drying rates rapidly reduced with reduction of MR until MR dropped down to about 0.1 for FBD, AUA and HWB and well below 0.1 for USP. As shown in Fig. 3, in general, among the drying methods employing FBD, USP had the highest drying rate, followed by HWB, and then FBD. It was interesting that AUA had the lowest drying rate among them, even though many researches pointing out that airborne ultrasound can enhance drying and reducing drying time [62], [63], [64]. In these previous studies, drying conditionsvaried a lot compared to this experiment, in terms of air velocity, drying methods and airborne ultrasound plate positions, etc. From this perspective, optimization of airborne ultrasound setups can be studied in further research. The higher drying rate for USP and HWB could result from the disruption of the cell structure of the seaweed samples bypower ultrasound [55] and blanching [65], which improved moisture diffusion within the seaweed matrix during drying.

**Fig. 5:**
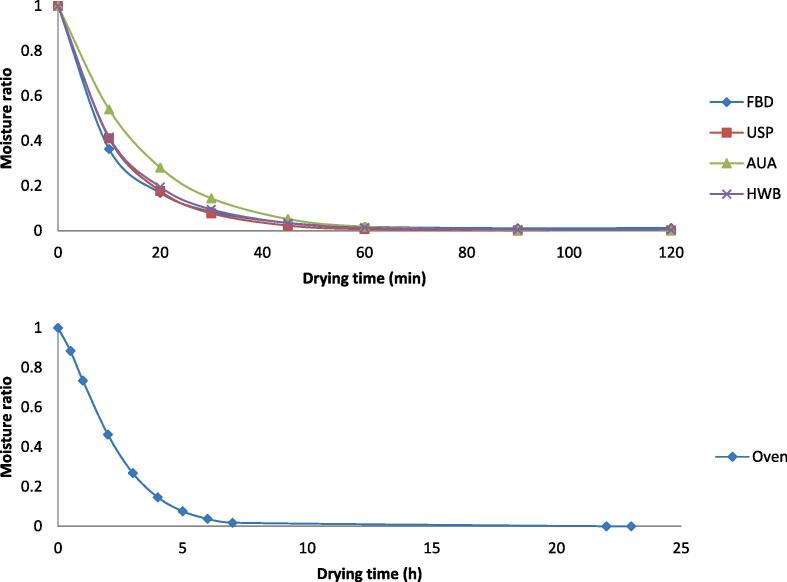
Drying kinetics of all drying methods in fitted modelsNote: all data are the means from 3 replicates.

Five drying kinetic models, as listed in Table 1, were used to investigate the drying methods. Parameters of the five drying kinetic models along with regression coefficients, RMSE, χ^2^, AIC and BIC are shown in Table 2.Wang and Singh model didnot show a good fit for the five drying methods in this experiment due to low R^2^ value. As for the other models they had similar R^2^ around 0.99, low RMSE from 0.0056 to 0.0189, AIC (<−26.92) and BIC (<−58.88). Page model fitted oven drying better due to its lowest χ^2^ (0.0001), AIC (−89.76) and BIC (−97.11), as similar results reported by Djaeni and Sari [66] studied on seaweed. The Page model was also employed in the present study to describe USP and AUA due to its smaller deviation at low MR points, which could predict the drying timemore accurately. The Page model was employed in previous literature for *Ascophyllum nodosum* [34], [67]. Midilliet al. model showed the best fit to FBD and HWB based on lowest RMSE. Mirzaee et al. [68] claimed that Midilli et al. model could satisfactorily fit apricot drying process. Kinetics models picked for all drying methods are shown in Fig. 5. The drying times to 10% moisture content predicted based on chosen models were 80 min for USP, HWB and FBD, 100 min and 6.5 h for AUA and oven drying respectively. Oven drying was much slower than other drying methods. Similarly, Moreira et al. claimed that it needed 5.5 h to dry Ascophyllum nodosum to 10% moisture content at 50℃by convective air drying (2 m/s) [69].

**Table 2:** Parameters and regression coefficient of the different models applied to drying kinetics of five drying methods.

Model	Parameter	FBD	USP	AUA	HWB	Oven
Newton	k	0.0938	0.0919	0.0673	0.0881	0.4214
	R^2^	0.998	0.999	0.998	0.998	0.991
	RMSE	0.0184	0.0134	0.0157	0.0186	0.0434
	X^2^	0.0004	0.0002	0.0003	0.0004	0.0021
	AIC	−57.556	−62.659	−60.1	−57.311	−60.006
	BIC	−63.956	−69.059	−66.5	−63.711	−69.006

Henderson and Pabis	a	0.9941	1.0023	1.0071	0.9992	1.0557
	k	0.0933	0.0921	0.0677	0.088	0.4459
	R^2^	0.9981	0.999	0.998	0.998	0.989
	RMSE	0.0182	0.0132	0.0154	0.0187	0.0392
	X^2^	0.0004	0.0002	0.0003	0.0005	0.0019
	AIC	−52.061	−57.228	−54.73	−51.678	−61.814
	BIC	−61.981	−67.149	−64.651	−61.598	−69.163

Page	k	0.0419	0.0116	0.0129	0.0125	0.0638
	n	1.0176	1.3941	1.2897	1.3436	1.9933
	R^2^	0.999	0.999	0.997	0.999	0.999
	RMSE	0.0116	0.0087	0.0189	0.0128	0.011
	X^2^	0.0002	0.0001	0.0005	0.0002	0.0001
	AIC	−59.339	−63.945	−51.497	−57.747	−89.761
	BIC	−69.26	−73.865	−61.417	−67.668	−97.111

Weibull	a	1.0054	1.0092	1.0127	1.0062	0.97672
	k	0.0429	0.0124	0.0141	0.0131	0.05271
	n	1.0119	1.3775	1.268	1.3323	2.10705
	R^2^	0.906	0.999	0.999	0.998	0.999
	RMSE	0.0079	0.0154	0.013	0.0178	0.01066
	X^2^	0.0001	0.0004	0.0003	0.0005	0.00016
	AIC	−56.158	−45.443	−48.117	−43.154	−85.234
	BIC	−73.097	−62.382	−65.056	−60.093	−95.506

Midilli et al.	a	1.0003	1.0004	1.0012	0.9996	0.9916
	k	0.1526	0.0753	0.0478	0.1091	0.2999
	n	0.8218	1.0768	1.1247	0.9048	1.337
	b	0.0001	0.0001	0.0001	0	−0.0001
	R^2^	0.999	0.998	0.999	0.999	0.999
	RMSE	0.0056	0.0153	0.0105	0.0127	0.0106
	X^2^	0.0001	0.0005	0.0002	0.0003	0.0002
	AIC	−43.037	−26.924	−32.93	−29.913	−78.002
	BIC	−74.99	−58.877	−64.883	−61.866	−91.955

Wang and Singh	a	−0.0262	−0.0259	−0.0235	−0.0251	−0.1779
	b	0.0002	0.0002	0.0001	0.0001	0.0059
	R^2^	0.773	0.815	0.846	0.824	0.939
	RMSE	0.1997	0.1843	0.1637	0.1738	0.1039
	X^2^	0.0532	0.0453	0.0357	0.0403	0.0132
	AIC	−13.774	−15.063	−16.957	−15.998	−40.384
	BIC	−23.694	−24.984	−26.878	−25.919	−47.733

Note: FBD, fluidized bed drying; USP, ultrasound pre-treatment; HWB, hot water blanching; Cl, confidence interval; RMSE, root mean square error; AIC, Akaike’s information criterion; BIC, Bayesian information criterion; all data are the means from 3 replicates.

Table 3 shows the energy consumption for each method studied. It can be seen that HWB and USP required 1.63 ± 0.02 and 1.58 ± 0.02 kWh respectively to complete the drying process. Energy consumption in HWB for its pre-treatment did not cover that for heating-up of the water bath prior to blanching, meaning less energy was considered in the present study. Among the studied drying methods, oven drying and AUA consumed energy the most. Long processing time of the oven method leadstohigh energy consumption. As for AUA, it was caused by the consumption by the airborne ultrasound generator, which could not reduce the drying time in the present experimental setup in addition to the energy consumption for drying. In general, FBD was a better choice in this study in terms of both energy consumption and process complexity.

**Table 3:** Energy consumption, drying time and product yield of dried seaweeds dried to 10% moisture content (w.b.) by the five drying methods.

	Energy (kWh)	Predicted Drying time (min)	Yield (g / 200 g fresh seaweed)
FBD	1.59 ± 0.13^a^	80	60.51 ± 1.00^a^
USP	1.63 ± 0.02^a,b^	80	49.70 ± 3.63^b^
HWB	1.58 ± 0.02^b^	80	50.89 ± 0.28^b^
AUA	2.47 ± 0.16^c^	100	58.17 ± 1.42^a^
Oven	2.51 ± 0.11^c^	390	55.16 ± 4.59^a,b^

Note: Data in the same column with the same letter are not significantly different (P > 0.05); all data are the means from 3 replicates.

Although HWB and USP demanded lower energy, their product yields were about 50 g dried seaweeds per 200 g fresh seaweeds, lower than the other methods by 5–10 g, as exhibited in Table 3. USP yield was lower significantly (P < 0.05) than FBD, AUA and oven. It is quite reasonable since solid loss occurred inthepre-treatments, especially for USP as the high power ultrasound cavitation in the pre-treatment would disrupt texture of the sample, resulting in insoluble and even some insoluble solids segment leachedout into the water. Solid lost for the pre-treatments were measured, they were7.65 ± 0.09 g and 6.32 ± 0.19 g for USP and HWB, respectively. FBD had the highest yield, followed by AUA, and then oven drying.

### 3.2 Water activity (*a_w_*) and colour

Water activities of fresh seaweedof ~0.99 were considerably decreased to between 0.19 and 0.37 after drying to 10% moisture content, as listed in Table 4. It is interesting to find that HWB and USP caused significantly (P < 0.05) lower water activity values than the other methods, by over 0.1. Water activities for the other methods varied from 0.321 to 0.373. The lower water activities in HWB and USP would attribute to the solid loss in their pre-treatments. Normally, food products with *a_w_* at 0.3 have the most stable status with regards to lipid oxidation, non-enzymatic browning, enzyme activity and the microbial parameters. However, froma preservation perspective, the water activity of dehydrated food only requires no more than 0.62 [70]. Hurdle technologies could be taken into consideration to combat the deleterious effects of seaweed [71]. And a higher value of*a_w_*of *Ascophyllum nodosum* could be allowed for long term preservation, and at the same time, the drying time, as well as the energy consumption, could be reduced.

**Table 4:** Water activities and colour values of seaweed after drying and after rehydration.

Samples	After Drying					After rehydration			
	*a_w_*	L*	a*	b*	ΔE*	L*	a*	b*	ΔE*
Oven	0.373^a^	26.58 ± 1.63^a^	−0.03 ± 0.01^a^	4.14 ± 0.28^a^	7.76 ± 0.69^a^	29.78 ± 0.01^a,c^	−3.18 ± 0.27^a^	14.97 ± 0.23^a^	4.66 ± 0.34^a^
FBD	0.351^b^	31.48 ± 0.98^a^	−1.00 ± 0.12^b^	5.99 ± 0.41^b^	6.52 ± 0.12^a^	28.79 ± 0.04^b^	−3.54 ± 0.27^a,b^	14.79 ± 0.58^a^	4.57 ± 0.59^a^
USP	0.193^c^	26.95 ± 2.03^a^	−0.53 ± 0.07^c^	4.49 ± 0.31^b^	7.40 ± 0.81^a^	31.40 ± 0.62^b,c^	−3.70 ± 0.02^a,b^	16.05 ± 1.12^a^	6.40 ± 0.97^a^
HWB	0.238^d^	27.93 ± 1.83^a^	−0.70 ± 0.03^b,c^	5.42 ± 0.30^b^	6.36 ± 0.54^a^	31.97 ± 0.19^c^	−4.23 ± 0.20^b^	16.83 ± 0.47^a^	7.49 ± 0.31^a^
AUA	0.321^e^	29.70 ± 4.40^b^	−0.87 ± 0.25^b,c^	5.33 ± 1.09^b^	7.39 ± 0.87^a^	28.49 ± 0.59^c^	−3.75 ± 0.04^a,b^	14.35 ± 1.00^a^	4.46 ± 0.57^a^

Note: Data in the same column with the same letter are not significantly different (P > 0.05); all data are the means from 3 replicates.

Colour of dried and rehydrated seaweed samples are listed in Table 4. Colour values L* (28.33 ± 2.23), a* (−0.31 ± 0.24) and b* (11.6 ± 1.33) of the defrost sample were also measured, based on which total colour changes (ΔE*) were calculated. As showed in Table 4, in general, after drying, L* values of the seaweed samples varied gently, a* decreased slightly, but b* considerably reduced. The huge reduction of b* caused large ΔE* (>6) for all the samples, among which samples dried by USP and AUA had higher values of ΔE* (>7.0) and the oven-dried sample had the highest (>7.7). ΔE* can be classified respectively as small difference when ΔE* ≤ 1.5, the distinct difference when 1.5 < ΔE* ≤ 3, and very distinct difference when ΔE* > 3 [72].This means that the dried sample’s colours differed distinctly from the defrost one. The highest ΔE* value for the oven-dried sample could belinked to the degradation of some pigments (i.e. carotenoids) during the long processing time [73]. Tekin et al., [74] pointed out that increase of a*, a decrease of b* of oven drying was related to browning reactions and degradation of the heat-stable green and yellowish pigments. The higher ΔE* values of AUA- and USP-dried samples indicated that ultrasound application could stimulate pigment degradation. It can be found also in the research by Tekin et al. [74]. After rehydration, compared to the defrosted sample, L* became higher, especially for samples with pre-treatment; a* values were much lower, indicating much stronger green colour, and their b* values were all higher, even though the values for dried samples were much lower than the defrosted one. Their ΔE* values were smaller than those for dried samples but still were over 3, indicating distinct different from the defrosted sample. Colour for the two pre-treatments methods after rehydration differed the most from the defrosted seaweed. They have more distinct variance in comparison with other methods.

**Fig. 6:**
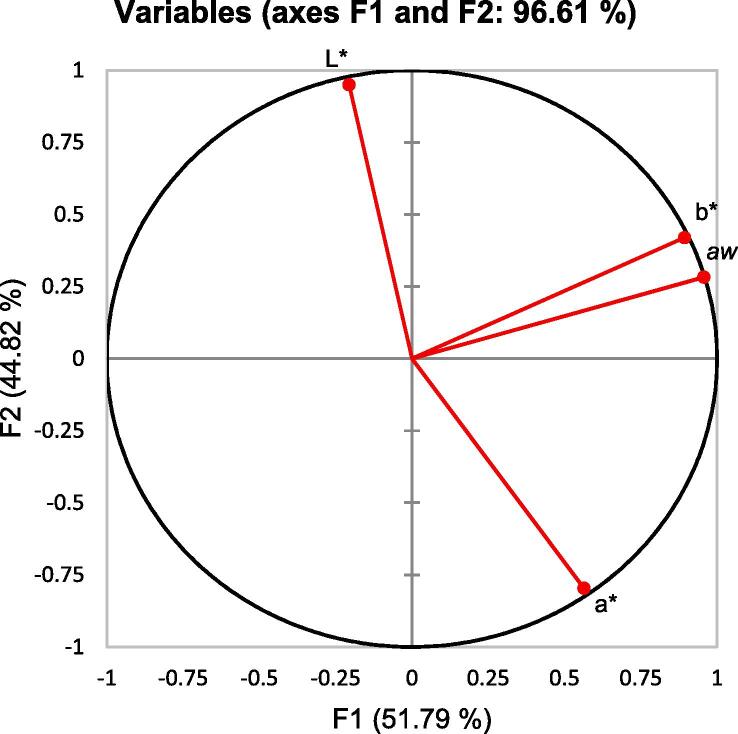
Correlation circle of a_w_, L*, a* and b* Note: all data are the means from 3 replicates.

To verify the correlation between water activity and colour of dried samples, the Pearson correlation analysis among *a_w_* and L*, a*, b* was applied and is reported in Table 5. Overall, b* value showed a high correlation coefficient with *a_w_* (r^2^ = 0.960). PCA was introduced to analyze the correspondence between aw and dried sample colour. Analyzed data were illustrated in the circle of correlations Fig. 6*.* The first two principal components (expressing 96.61% of initial variances of *a_w_* and L*, a*, b* were selected to evaluate the correlation. The first component (F1, 51.79%) was expressed dominantly by *a_w_* and b* value which showed that the yellowness of dried seaweed was strongly correlated with *a_w_*.

**Table 5:** Pearson’s correlation coefficients of a_w_, L*, a* and b*

Variables	*a_w_*	L*	a*	b*
*a_w_*	1.000	0.087	0.329	0.960
L*		1	−0.822	0.177
a*			1	0.134
b*				1

Note: all data are the means from 3 replicates.

### 3.3 Rehydration capacity

Seaweed is oftendried to increase shelf life. Dried seaweeds are commonly rehydrated before consumption in order to recover some of the properties, and thatrehydration capacity is one of the quality parameters toward different drying technologies. Rehydration is composed of three simultaneous steps: water absorption, swelling and soluble compounds diffusion [75]. It is generally accepted that the degree of rehydration is dependent on the degree of cellular and structural disruption. After drying, there can be cellular rupture and dislocation, resulting in a dense structure of collapsed as shown in Fig. 8. The moisture contents of dried samples in this study, listed in Table 6, exceeded defrost sample’s by 3–6% after one-hour rehydration. This fact could be explained by the loss of solids of the seaweed during the rehydration process, which could lead to more porous seaweed structure to be replaced by water, resulting better hydrophilic properties [34], [67]. USP and HWB samples showed higher moisture content than other drying methods. During the pre-treatment processes, the solid matrix of the samples was disrupted, which could cause water easier and faster to diffuse into the seaweed samples during the rehydration process. Oven, FBD and AUA have similar rehydration ability.

**Table 6:** Moisture content of defrost sample and rehydrated samples.

Samples	Moisture content (%)
defrost	73.08 ± 0.68
Oven	76.44 ± 1.39^a^
FBD	76.33 ± 0.33^a^
USP	79.00 ± 1.18^a,b^
HWB	79.27 ± 0.87^b^
AUA	77.38 ± 0.90^a,b^

Note: Data in the same column with the same letter are not significantly different (P > 0.05); all data are the means from 3 replicates.

### 3.4 Total phenolic content (TPC)

**Fig. 7:**
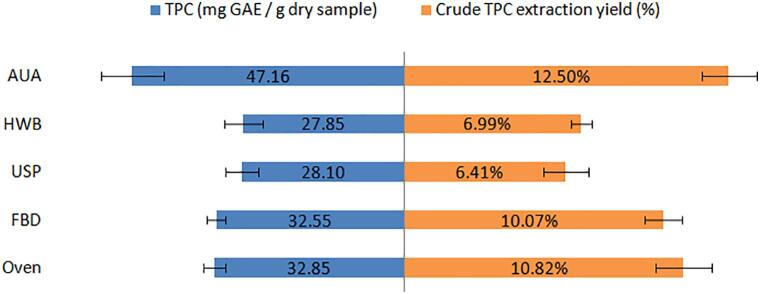
TPC and crude extractionyieldforthefive drying methods *Note: all data are the means from 3 replicates.*

Crude extraction yields from 2 g of dried samples areshown in Fig. 7 for the five drying methods. The extraction yields for oven and FBD were similar, indicating fluidized bed drying would not affect the extraction yield. Due to the solid loss during the pre-treatments, HWB (6.99 ± 0.39%) and USP (6.41 ± 0.86%) only had 50% of yield in comparison with AUA. On the other hand, AUA dried samples hadthe highest yield (12.50 ± 1.03%). Yuan et al. [76] employed microwave-assisted extraction to extract antioxidant compounds from *Ascophyllum nodosum*. The extraction yield was enhanced, from 10.41% by conventional methanol extraction, to 12.46% by the novel extraction technology. In the present study, AUA achieved similar enhancement effect, increasing the extraction from 10.82% (for oven drying) to 12.50%, indicating AUA could improvethe extractability of phenolic compounds from seaweeds, although the objective of the irradiation of airborne ultrasoundwas not initially intended for extractionpurposes. The higher extraction yield for the samples dried by using AUA couldbe caused by ultrasonication generated cavitation and/or micro-channels, which increases the extractability of phenolic compounds by increasing the disruption of the seaweed cells structure.

**Fig. 8:**
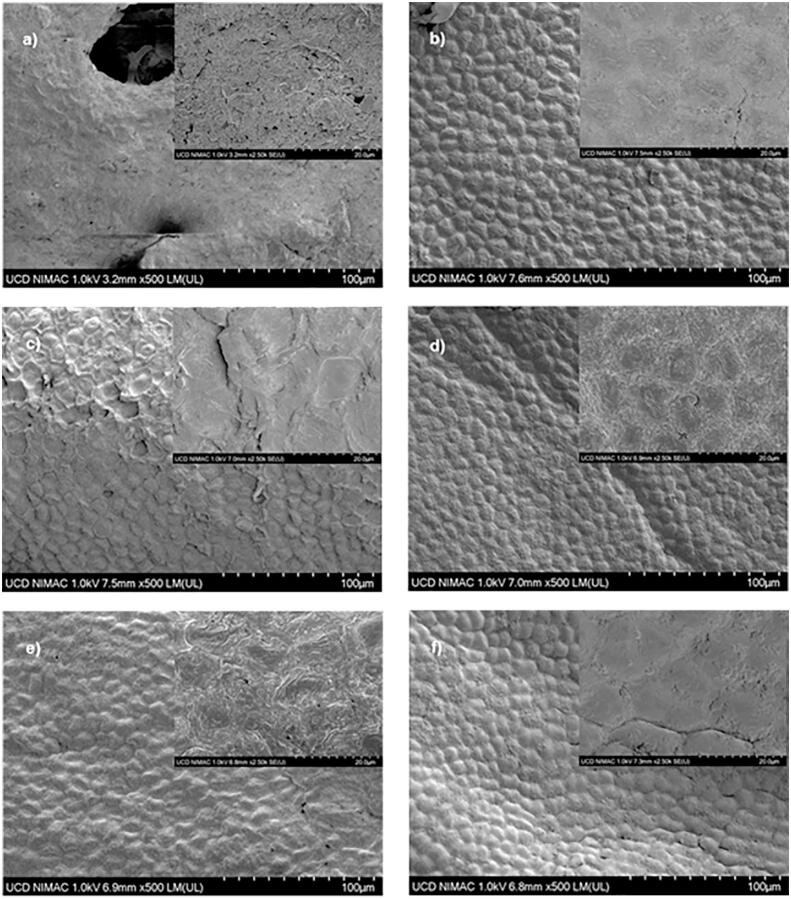
Microscopic structure of the *Ascophyllum nodosum* samples: a) after defrosting, and after rehydration of samples dried byb) Fluidized bed drying, c) oven drying,d) ultrasound pre-treated fluidized bed drying, e) airborne ultrasound-assisted fluidized bed drying and f) hot water blanching pre-treated fluidized bed drying.

In the present study, TPC values varied from 27.85 to 47.16 GAE/g dried samples. This result was similar to those reported by Moreira et al. [69]. According to their study, 50 °C convective air-dried *A. nodosum* contained 31.8 ± 1.31 mg phloroglucinol equivalent /g of dry sample. However, TPC in this study was much higher than that reported by Sabrina et al. [77], where TPC was 12.1 ± 0.2 mg GAE/g oven-dried Irish brown seaweed at 40 °C for 24 h. The variation may be caused by the harvest season and geographical conditions [78].

As demonstrated in Fig. 7 oven and FBD dried samples had similar TPC (32.85 ± 2.10 and 32.55 ± 1.81 mg GAE/g dried sample, respectively). HWB (27.85 ± 3.2) and USP (28.10 ± 2.81) samples had the lowest TPC, as expected, mainly due to the solid loss as discussed above. TPC in AUA dried samples (47.16 ± 5.73 mg GAE/g dried sample) was significantly higher than other methods (P < 0.05). This may predominantly result from the much higher extraction yield as aforementioned. Other researchers also pointed out that airborne ultrasound could cause the change of microstructure and increase extractability of many bioactives such as total phenolic compounds and vitamins [63], [79]. Rodríguez et al. [80] reported airborne ultrasound could reduce TPC loss during convective drying of apple at low drying temperature (30 °C), in comparison with convective drying without ultrasound. It is to say, convective drying with airborne ultrasound resulted in higher TPC levels in the dried apple than convective drying without ultrasound. However, in their study, the effect of ultrasound depended on the drying temperature. A higher drying temperature (50 or 70 °C) caused alower TPC in apple dried with ultrasound.

### 3.5 Total viable count (TVC)

The total viable count was carried out for all samples to assess the microbial load. In fresh samples (before any washing or treatment) the highest microbial count of 2.89 log_10_ was observed. After washing and grinding, the microbial load showed a reduction of 0.35 log_10_. After the final processing steps (freezing, respective drying treatments) microbial load was reduced to below the detection limit (moulds were not tested in present study). Although air drying at low temperature (50 to 60 °C) alone is not an effective method to inactivite microbes, dehydration process may affect cellular components, induce DNA and RNA breakdown, protein denaturation and cell wall damage [81].

### 3.6 SEM analysis

SEM analysis was carried out to explore the effect of different drying methods from seaweed cell structure perspective. SEM observations of the seaweed samplesat different magnifications (500× and 2500×) are presented in Fig. 8 *Ascophyllum nodosum* often has epidermal shedding, which is devoid ofcell contents, removable from the thallus surface [82], [83]. The shedding covered the defrosted seaweed sample, as shown in Fig. 8a, while Fig. 8b-f exhibits clear cellular structure. In the oven-dried sample, as illustrated in Fig. 8c, there was still some detritus of the shedding left on the thallus surface, while in other samples dried by methods involving FBD, few detritus could be seen. This indicates that the shedding at the surface of the thallus would be removed under the drying conditions of FBD, which would be the high speed of blowing air in the dryer. In Fig. 8f, a few tiny fissures can be seen and shallow pits at the cellular structure in the HWB dried sample, which would be caused by solid lost during blanching. As in Fig. 8d-e, there existed a similar pattern in the larger magnifications, which was distinguished from the others. The samples for these two pictures were subjected to ultrasound, in which Fig. 8d is for power ultrasound pre-treatment in water, and Fig. 8e for irradiation by airborne ultrasound during drying. These patterns would be related to the effect of ultrasound, such as cavitation or sponge effect. Drying methods can affect samples cell structure and porosity significantly [84]. Cárcel et al. [85] and Ozuna et al. [86] reported airborne ultrasound-assisted convective drying could generate micro-channels in their apple samples, caused by “sponge effect”. However, in the present study, only surface SEM was performed, and the matrix structure could not be seen. In order to identify the effect of ultrasound, a further SEM should be conducted in future to observe the cross-section of the samples.

## 4 Conclusions

In this study, a quantitative analysis of the drying kinetics and the product quality indicators of dried *Ascophyllum nodosum* were performed for five novel drying methods. All methods involving FBD performed better than oven drying in drying time, quality control and energy consumption. Moreover, FBD had a better production yield than other methods with comparable drying time. AUA showed better retention of total phenolics in *Ascophyllum nodosum*. On the other hand, both pre-treatment technologies were superior in relation to energy and drying time, exhibiting more efficiency comparing with other methods. The mechanism of the drying methods should be investigated further by examining the microstructure of cross-sections oftreated samples. In order to explore and optimize ultrasound-assisted drying for practical applications, further research and development of this technology is required.

## Declaration of Competing Interest

The authors declare that they have no known competing financial interests or personal relationships that could have appeared to influence the work reported in this paper.
